# Dietary total antioxidant capacity and head and neck cancer: a large case-control study in Iran

**DOI:** 10.3389/fnut.2023.1226446

**Published:** 2023-09-27

**Authors:** Fatemeh Toorang, Monireh Sadat Seyyedsalehi, Bahareh Sasanfar, Hamideh Rashidian, Maryam Hadji, Elham Mohebbi, Roya Safari, Farid Najefi, Ahmad Naghibzadeh-Tahami, Paolo Boffetta, Kazem Zendehdel

**Affiliations:** ^1^Cancer Research Center, Cancer Institute, Tehran University of Medical Science, Tehran, Iran; ^2^Departments of Medical and Surgical Sciences, University of Bologna, Bologna, Italy; ^3^Nutrition and Food Security Research Center, Shahid Sadoughi University of Medical Sciences, Yazd, Iran; ^4^Department of Nutrition, School of Public Health, Shahid Sadoughi University of Medical Sciences, Yazd, Iran; ^5^Health Sciences Unit, Faculty of Social Sciences, Tampere University, Tampere, Finland; ^6^Department of Oncology, Lombardi Comprehensive Cancer Center, Georgetown University, Washington, DC, United States; ^7^Research Center for Environmental Determinants of Health, School of Public Health, Kermanshah Medical Sciences University, Kermanshah, Iran; ^8^Social Development and Health Promotion Research Center, Kermanshah University of Medical Sciences, Kermanshah, Iran; ^9^Modeling in Health Research Center, Institute for Futures Studies in Health, Kerman University of Medical Sciences, Kerman, Iran; ^10^Health Foresight and Innovation Research Center, Institute for Futures Studies in Health, Kerman University of Medical Sciences, Kerman, Iran; ^11^Stony Brook Cancer Center, Stony Brook University, Stony Brook, NY, United States; ^12^Department of Family, Population and Preventive Medicine, Renaissance School of Medicine, Stony Brook University, Stony Brook, NY, United States; ^13^Cancer Biology Research Center, Cancer Institute, Tehran University of Medical Science, Tehran, Iran

**Keywords:** head and neck cancer, dietary antioxidant capacity, case–control study, FRAP, TRAP

## Abstract

**Background:**

Data on the association between head and neck cancer (HNC) and dietary factors are inconclusive. No study has so far investigated the association between dietary total antioxidant capacity (dTAC) and HNC concerning interactions with other risk factors.

**Method:**

Pathologically confirmed new diagnosed HNC patients were included in this study. The control group was healthy hospital visitors who were frequently matched with patients on age (5 years interval), gender, and province of residence. Trained interviewers administered a validated Food Frequency Questionnaire (FFQ) to assess the participants’ food intake 1 year before the cancer diagnosis. Data on TAC scores of foods was collected by Ferric Reducing Antioxidant Power (FRAP) and Total Radical-trapping Antioxidant Parameters (TRAP) from published data. We applied logistic regression adjusted for age, sex, energy intake, socioeconomic status, province, opium use, alcohol use, physical activity, and dental health. We also studied the interaction of dTAC with tobacco smoking status, and opium use on the risk of HNC.

**Results:**

We recruited 876 HNC patients and 3,409 healthy controls. We observed a significant decrease in the odds of HNC with increasing dTAC scores. The OR of HNC for the third vs. the first tertile was 0.49 (95%CI 0.39–0.61) for FRAP and 0.49 (95%CI 0.39–0.62) for TRAP. Both dTAC scores were inversely associated with lip and oral (T3 ver. T1 OR = 0.51; 95%CI 0.36–0.71 for FRAP and OR = 0.59; 95% CI 0.44–0.82 for TRAP) and larynx (T3 ver. T1 OR = 0.43; 95%CI 0.31–0.61 for FRAP and OR = 0.38; 95% CI 0.26–0.55 for TRAP) cancers. There was no interaction between tobacco smoking, opium use; and TRAP or FRAP on the risk of HNC.

**Conclusion:**

An antioxidant-rich diet in terms of FRAP or TRAP could decrease the risk of HNC and its subtypes.

## Highlights

- Data on the association between head and neck cancer (HNC) and dietary factors are inconclusive.
- An antioxidant-rich diet in terms of FRAP or TRAP could decrease the risk of HNC and its subtypes.
- Promoting consumption of high antioxidant foods such as fruit, vegetable, and whole grains may reduce the risk of HNC.

## Background

Head and Neck Cancers (HNC) are the seventh most common cancer in the world, and these cancers account for 4% of cancer deaths worldwide (1, 2). In addition to the psychological problems of the patients and their families, disability, and death caused by HNC impose a tremendous economic burden on our society (3–5). Sixty-five percent of HNC cancers occur in low and middle-income countries (6). Unfortunately, the prognosis of HNC is poor (7) and survivors struggle with various problems, including breathing and eating disorders (8). Therefore, identifying risk factors and implementing prevention are the primary strategies for controlling these cancers.

Several risk factors are associated with HNC, including cigarette smoking, alcohol drinking, asbestos exposure, HPV infection, and opium use (6, 9) A recent comprehensive review of nutritional factors and cancer risk by the World Cancer Research Fund (WCRF) and the American Cancer Institute concluded that data on associations between several dietary components and HNC are inconclusive (6). This review concluded for limited evidence of a protective effect of non-starchy vegetables and a healthy nutritional lifestyle against HNC. No definitive conclusions can be drawn regarding other dietary factors, including fruits, legumes, animal foods, and other nutrients (6).

Chronic inflammation, shown by increased levels of inflammatory cytokines, plays an essential role in developing various types of cancer, including HNC (10, 11). Dietary factors can inhibit or accelerate inflammation in our bodies (12). Dietary components such as phenols, antioxidant vitamins, and inflammatory nutrients were associated with cancer (13). Also, consuming several foods, including fruits and vegetables or animal foods, can influence inflammatory pathways that alter cancer development (12, 14, 15). Some food factors increase inflammatory cytokines and endothelial growth factors, which increase angiogenesis and proliferation (15–17). Several studies have investigated the effects of these inflammatory factors on HNC development (10, 16). On the other hand, several studies have shown that consuming fruits, chocolate, berries, whole grains, and other antioxidant-rich foods can reduce cancer incidence (6, 18, 19). It is claimed that dietary antioxidants can reduce reactive oxygen or nitrogen species, thereby reducing DNA oxidation (11, 13).

Previous studies examined the association between one or more foods or nutrients and the risk of different cancers separately (20). However, dietary components are not eaten separately, and the interactions between different foods may increase or decrease the risk of different diseases (21). Therefore, the effect of the whole diet on disease risk will differ from each component alone (21). To consider this, total dietary antioxidant capacity (dTAC) scores were introduced to calculate the antioxidant effects of whole diets (22–24). Several studies have examined the association between these dietary scores and colon, breast, and endometrial cancer risk (14, 25, 26). Data on the association between HNC and nutritional components are limited, and the existing reports are inconclusive. In this study, we evaluated for the first time the association between dTAC and HNC in a large case–control study.

## Methods

This study was conducted using data from the IROPICAN study. That study is a large multicenter case–control study that evaluated the relationship between opium use and the risk of lung, bladder, head and neck, and colorectal cancers. The data has been collected in 10 provinces in the east, south, north, and center of Iran (27). The participants were interviewed using standard questionnaires about their lifestyle and validated Food Frequency Questionnaire (FFQ) (28).

Pathologically confirmed incident HNC patients with no cancer history were included in this study. Tumor subsite was uniformly classified using ICD-O3 codes. HNC cases in this study included ICD-O3 HNC codes of oral, larynx, and pharynx (e.g., C00–C09, C11, C12, C14, C31, and C32). The control group was healthy hospital visitors, frequency matched on age (5 years apart), gender, and province of residence. Controls were independently matched to the patient group of each cancer type, but we used control data from all cancer types for our analysis. They were relatives or friends of non-cancer patients and were visiting the general hospitals where the cases were selected (29). The main inclusion criteria for patients was pathologically confirmed HNC. The exclusion criterion was being affected by other cancers rather than HNC. In controls, exclusion criterion was diagnosis of any cancers.

Trained interviewers conducted the face-to-face interview using a structured questionnaire to obtain demographic and other non-dietary data. A detailed description of the questionnaire and computing-related scores are described previously (16). Socioeconomic status (SES) was determined using principal component analysis by combining some data related to education, income, and ownership of some home appliances. Then we put subjects in three groups based on scores in control group. Physical activity workload (PPWL) was estimated based on job history of participants using the Finland Job Exposure Matrix (FINJEM) (30). Then, the participants were categorized to three groups of physical activity based on PPWL scores: sedentary (zero PPWL-years), moderate (PPWL-years greater than zero and less than or equal to 4.80), and heavy (PPWL-years greater than 4.81). Dental health was defined by a score in our study considering decayed, missing and filled teeth, participants were categorized to three categorizes based on scores in control group.

### dTAC scoring

Trained interviewers administered validated FFQs to assess the participants’ food intake a year before the study (31). Long term dietary habits of individual are associated with their health outcomes. As HNC extremely affects patients’ food habits, we asked patients to report their food intake before their cancer diagnosis. All study data, including dietary data, were entered into an online system that supervisors could continuously review. The food composition table was prepared using USDA food composition tables (32). Data on TAC scores of foods by Ferric Reducing Antioxidant Power (FRAP) and Total Radical-trapping Antioxidant Parameters (TRAP), which measure the reducing power and the chain-breaking antioxidant capacity, respectively, was gathered from published data (33, 34).

### Statistical analysis

The TRAP and FRAP scores were adjusted for energy by the residual method suggested by Willett (35). Before analyzing the data 76 subjects were omitted because of incomplete questionnaires or pathological issues (unconfirmed pathological diagnosis or metastases). Subjects who reported energy intake less than 500 and over 4,500 Kcal/d were considered outliers and omitted. It caused a 2% reduction in both cases and controls (18 subjects out of 849 patients and 74 subjects out of 3,484 controls). Participants were classified into tertiles of dTAC scores based on the distribution in the control group. We applied unconditional logistic regression in crude and adjusted models to determine the association between dTAC scores and HNC. The full model was adjusted for age (5categories), sex(male/female), province of residence (10 provinces), energy intake (kcal/day, continuous), socioeconomic status (low, medium, high), opium use (yes, no), tobacco smoking (yes, no), water pipe smoking (yes, no) regular alcohol drinking (yes, no), physical activity (sedentary, moderate, heavy, unknown) and dental health (poor, moderate, good). We tested the linear trend by treating the median of TRAP or FRAP scores in each tertile as a continuous variable. An interaction term was added to models to analyze the interaction between anti-oxidant activity and smoking status, opium uses and physical activity, and the risk of HNC. The *p*-value for interactions was estimated by the likelihood ratio test between the models with and without the interaction term. Subjects were categorized to five groups based on their history of tobacco use: current users, occasional users, quitting tobacco use for less than 10 years, quitting tobacco use for more than 10 years and never users. The heterogenicity of the association between TRAP or FRAP scores and risk of HNC in different groups of tobacco use was analyzed. All statistical analysis was done in STATA software (Stata 14.1, College Station, Texas 77,845 USA). Two-sided *p* values < 0.05 were considered statistically significant.

## Results

Totally 879 patients and 3,409 healthy controls were recruited for our study. Almost two third of participants were male in both groups (Table 1). Tobacco smoking and opium use were less common in controls. Regular alcohol use was uncommon in both groups. Energy and food group intakes were not significantly different between patients and controls (Table 2).

**Table 1:** Characteristics of head and neck cancer patients and controls in the IROPICAN study.

Characteristics		Head and neck cancer patients *N* (%)	Controls *N* (%)
Total		876	3,409
Gender	Male	662 (76)	2,356 (69)
	Female	214 (24)	1,053 (31)
Age group	30–39	59 (6.7)	250 (7.3)
	40–49	135 (15.4)	548 (16.1)
	50–59	265 (30.3)	1,055 (30.9)
	60–69	287 (32.8)	1,071 (31.4)
	≥70	130 (14.8)	485 (14.2)
SES^1^	Low	342 (39)	953 (28)
	Median	290 (33)	1,141 (33)
	High	244 (28)	1,315 (39)
Opium use	No	474 (54)	2,954 (87)
	Yes	402 (46)	455 (13)
Smoking	No	382 (44)	2,448 (72)
	Yes	494 (56)	961 (28)
Water pipe use	No	796 (91)	3,185 (93)
	Yes	80 (9)	224 (7)
Regular alcohol use	No	805 (92)	3,268 (96)
	Yes	71 (8)	141 (4)
PPWL^2^	Sedentary	267 (30)	1,104 (32)
	Moderate	200 (23)	746 (22)
	Heavy	202 (23)	749 (22)
	Unknown	207 (24)	810 (24)
Dental health^3^	Poor	207 (24)	1,551 (46)
	Moderate	75 (9)	449 (13)
	Good	594 (68)	1,409 (41)
Province	Tehran	157 (17.9)	813 (23.9)
	Fars	376 (42.9)	933 (27.4)
	Kerman	156 (17.8)	523 (15.3)
	Golestan	36 (4.1)	369 (10.7)
	Mazandaran	17 (1.9)	134 (3.9)
	Kermanshah	37 (4.2)	250 (7.3)
	Khorasan-Razavi	29 (3.3)	155 (4.5)
	Bushehr	0 (0)	67 (1.9)
	Hormozgan	33 (3.7)	78 (2.3)
	Systan-Balouchestan	36 (4.0)	100 (2.9)

1 SES: socio-economic status was determined using principal component analysis by combining some data related to education, income, and ownership of some home appliances.

2 Physical activity workload (PPWL) was estimated based on the Finland Job Exposure.

3 Dental health was defined by DMFT score sum of the number of decayed, missing and filled teeth.

**Table 2:** Dietary intake of head and neck cancer patients and controls in IROPICAN study.

Food group gram/day (Mean ± SD)	Head and neck cancer patients (*n* = 876)	Controls (*n* = 3,409)
Energy	1845.9 ± 796.6	1850.6 ± 682.9
Cereal	500.9 ± 258.0	493.1 ± 268.7
Vegetables	439.8 ± 407.7	442.8 ± 243.6
Fruits	357.5 ± 269.9	362.7 ± 258.7
Nuts	5.9 ± 7.6	5.9 ± 9.3
Legumes	31.6 ± 39.8	32.1 ± 27.5
Vegetable oils	17.2 ± 13.4	17.8 ± 14.2
Confectionary	38.7 ± 38.2	38.3 ± 37.9
Total meat	66.9 ± 47.8	68.7 ± 50.8
Red mea	22.2 ± 24.2	23.6 ± 28.3

The contribution of food groups to dTAC scores is shown in Table 3. Cereals were the main provider of antioxidants in our study (37.2% for FRAP and 53.9% for TRAP). Other sources of FRAP and TRAP in our control group were fruits (32.6 and 8.3%), vegetables (19.6 and 31.7%), legumes (4.7 and 1%), nuts (3.3 and 3.8%), confectionaries (1.1 and 1.1%), and fruit juice (3.6 and 1.0%).

**Table 3:** Correlation and contribution of food groups to overall dTAC intake among 3,409 controls in the IROPICAN study.

		Cereals	Fruits	Vegetables	Legumes	Nuts	Confectionaries	Juices
TRAP^1^	Correlation^3^ R (95%CI)	0.51 (0.5–0.53)	0.07 (0.06–0.07)	0.38 (0.36–0.39)	0.005 (0.004–0.006)	0.03 (0.02–0.03)	0.006 (0.004–0.007)	0.005 (0.003–0.007)
	Contribution Percent(SD)	53.9 (17.2)	8.3 (6.5)	31.7 (14.5)	1.0 (1.1)	3.8 (4.1)	1.1 (1.7)	1.0 (2.6)
FRAP^2^	Correlation^4^ R (95%CI)	0.21 (0.19–0.22)	0.5 (0.49–0.52)	0.18 (0.17–0.18)	0.03 (0.02–0.03)	0.05 (0.04–0.05)	0.01 (0.01–0.01)	0.05 (0.04–0.05)
	Contribution Percent(SD)	37.2 (16.5)	32.6 (15.8)	19.6 (9.7)	4.7 (4.0)	3.3 (4.8)	1.1 (1.8)	3.6 (6.3)

1 FRAP: Ferric Reducing Antioxidant Power of the diet.

2 TRAP: Total Radical-trapping Antioxidant Parameters of the diet.

^3,4^The Pearson’s correlation coefficient for all these food groups was significant at *p* < 0.05 level.

We observed a significant decrease in the OR of HNC with increasing dTAC scores (Table 4). Compared with the lowest tertile, the OR of HNC for the third tertile decreased by 51% for both FRAP (OR 0.49; 95%CI 0.39–0.61) and TRAP (OR 0.49; 95%CI 0.39–0.62). When we investigated subtypes of HCN cancers, a significant decrease in the risk of lip and oral cavity cancer was seen by higher FRAP (OR 0.51; 95%CI 0.36–0.71 for the third tertile compared to the first, *P*trend < 0.001) and TRAP (OR 0.59; 95%CI 0.44–0.82 for the third tertile compared to the first, *P*trend = 0.003). Considering larynx cancer, a 57% reduction in risk was observed in the third tertile of FRAP compared to the first tertile (OR 0.43; CI 95% 0.31–0.61, *P* trend<0.001). The corresponding figure was 62% for the TRAP score (OR 0.38; 95% CI 0.26–0.55, *P* trend < 0.001). The difference in association between lip and oral cavity and laryngeal cancer with FRAP (*p* = 0.72) and TARP (*p* = 0.14) was not statistically significant Moreover, the association of head and neck cancers with FRAP and TARP (*p* = 0.38) did not differ significantly. There was no interaction between TRAP or FRAP scores with cigarette smoking, opium use, tobacco and water pipe use, physical activity, gender and age in our study (Supplementary Table 1). The differences in association between TRAP or FRAP scores and risk of HNC in different group of tobacco use was not significant.

**Table 4:** The association of dietary Total Antioxidant Capacity (dTAC) scores with head and neck squamous cell carcinoma and its subtypes in the IROPICAN study.

			First tertile	Second tertile	Third tertile	*p* trend^1^	OR and 95%CI (continuous)
FRAP^3^	All HNSCC	Case/Control	361/1138	261/1136	254/1135	-	-
		Crude	Reference	0.72 (0.61–0.87)	0.70 (0.59–0.84)	<0.001	0.74 (0.66–0.83)
		Adjusted^2^	Reference	0.63 (0.51–0.77)	0.49 (0.39–0.61)	<0.001	0.74 (0.66–0.83)
	Lip and oral cavity (313/3409)	Case/Control	147/1138	90/1136	76/1135	-	-
		Crude	Reference	0.61 (0.47–0.81)	0.52 (0.39–0.69)	<0.001	0.74 (0.63–0.86)
		Adjusted	Reference	0.62 (0.46–0.84)	0.51 (0.36–0.71)	<0.001	0.72 (0.60–0.86)
	Larynx (427/3409)	Case/Control	161/1138	129/1136	137/1135	-	-
		Crude	Reference	0.8 (0.63–1.03)	0.85 (0.67–1.09)	0.188	0.92 (0.81–1.03)
		Adjusted	Reference	0.62 (0.44–0.86)	0.43 (0.31–0.61)	<0.001	0.69 (0.58–0.82)
TRAP^4^	All HNSCC	Case/Control	390/1137	234/1137	252/1135	-	-
		Crude	Reference	0.60 (0.50–0.72)	0.64 (0.54–0.77)	<0.001	0.78 (0.71–0.87)
		Adjusted	Reference	0.49 (0.39–0.61)	0.49 (0.39–0.62)	<0.001	0.69 (0.60–0.79)
	Lip and oral cavity (313/3409)	Case/Control	109/1137	94/1137	110/1135	-	-
		Crude	Reference	0.86 (0.65–1.15)	1.0 (0.77–1.3)	0.938	0.96 (0.83–1.1)
		Adjusted	Reference	0.59 (0.43–0.81)	0.59 (0.44–0.82)	0.003	0.76 (0.63–0.92)
	Larynx (427/3409)	Case/Control	210/1137	106/1137	111/1135	-	-
		Crude	Reference	0.5 (0.39–0.65)	0.53 (0.41–0.68)	<0.001	0.72 (0.63–0.84)
		Adjusted	Reference	0.39 (0.28–0.55)	0.38 (0.26–0.55)	<0.001	0.59 (0.48–0.75)

The association between lip and oral cavity and laryngeal cancer with FRAP (*p* = 0.72) and TARP (*p* = 0.14) was not different. The difference between the association of head and neck cancers with FRAP and TARP (*p* = 0.38) was not significant.

1 Analyzed by using the median value of TRAP or FRAP as a continuous variable in unconditional logistic models.

2 Adjusted for age (5 categories), sex(male/female) and energy intake (kcal/day, continues), socioeconomic status (low, medium, high), province(10 different provinces), opium use (yes, no), smoking (yes, no), water pipe use (yes, no) regular alcohol use (yes, no), physical activity (sedentary, moderate, heavy, unknown) and, dental health(poor, moderate, good).

3 FRAP: Ferric Reducing Antioxidant Power of the diet.

4 TRAP: Total Radical-trapping Antioxidant Parameters of the diet.

## Discussion

This study found that dTAC scores in terms of FRAP and TRAP are inversely associated with HNC risk. No interaction was found between gender, tobacco smoking, opium use, water pipe use, physical activity, and dTAC scores in determining the risk of HNC.

These scores assess the overall antioxidant capacity of the diet, which adds value to the simple analysis of individual antioxidants or food items. Several studies showed an inverse association between the dTAC and the risk of colon, rectum, breast, and gastric cancers (14, 25, 26). Although the association between HNC cancer and dTAC was not studied previously, several studies showed inverse associations between individual antioxidants such as vitamin C, selenium, carotenoids, retinol, and vitamin E intake and the risk of HNC (8, 19).

Generally, fruit and vegetables are considered the primary source of antioxidants. They were the second and third contributors of dTAC in our study. Several studies showed that the intake of antioxidant reach foods such as vegetables or fruit is associated with the risk of HNC (18, 36). The main contributor to dTAC in our population was cereals. It seems that this high contribution is mainly due to the high consumption of cereals in our society as the staple food of Iranian (37). However, limited studies showed an inverse association between grain intake and the risk of HNC (38). Assessing the association between dTAC scores with the risk of HNC allows us to highlight the importance of consuming antioxidants from different sources and not just focusing on fruit and vegetables.

As mentioned above, several studies have shown a significant association between individual antioxidant intake and the risk of HNC (19). No study investigated the association between dTAC and the risk of HNC, but several studies found an inverse association between healthy dietary patterns and the risk of HNC (39, 40). Other studies found a robust association between adherence to healthy dietary patterns and dTAC score and health outcomes, including several cancers (24, 41). The association between dietary pattern and scores with HNC emphasized the importance of the intake of a wide range of antioxidants through adherence to healthy dietary patterns rather than focusing on some individual dietary antioxidants or supplements.

Several studies showed that intake of a diet low in antioxidants accompanied by smoking could exacerbate the carcinogenesis effects of tobacco. Smoking and alcoholic drinks have both been linked with increased oxidative stress (42, 43). Analyses of the International Head and Neck Cancer Epidemiology (INHANCE) consortium showed that the risk of HNC in subjects with a low intake of carotenoids and high exposure to smoking is 30 times higher than in subjects with a high intake of carotenoids and no alcohol and smoking exposure (44). Moreover, studies found high intake of flavonoids could decrease the risk of cancer, particularly in smokers (45). In our study higher dTAC score was associated with a lower risk of HNC both in smokers and non-smokers.

Other sources of antioxidants and oxidants like endogenous production in body could affects the association between antioxidant intake and risk of HNC cancer. Several studies showed the association between the endogenous antioxidant level such as serum levels of them with risk of HNC (46–48). Serum antioxidant enzymes levels including catalase (CAT), superoxide dismutase (SOD), glutathione peroxidase (GPx), and malondialdehyde (MDA) concentrations in blood are lower in cancer patients and are associated with cancer prognosis (48) and several studies proposed prescription of antioxidants rich products to decreasing the treatment adverse effects or slowing the cancer progress (49–51).

This study has several strengths. First, we used a large sample based on a multicenter study, allowing us to study the association of dTAC for HNC overall and by its subsites, including oral cavity and laryngeal cancers. The patients were pathologically confirmed by specialist. We also adjusted for several confounding variables, including tobacco smoking, and opium use. Using a validated FFQ was the second strength of our study (31). Moreover, the interviews were conducted in the same setting in all provinces by trained interviewers. However, there are some limitations to this study. Some scientists believe that TRAP and FRAP do not cover all the antioxidant power of the diet, particularly when computed using FFQ (52). However, several studies showed a strong association between these scores and the total antioxidant capacity of a diet and the serum antioxidant level of healthy adults (23, 52). Moreover, several studies in Iran and other countries showed a strong association between these scores and healthy dietary patterns such as healthy eating index (HEI), Mediterranean diet, and Dietary approach to stop hypertension (DASH) diet (22, 52, 53). The positive association between dTAC scores and fruit and vegetable consumption, along with the negative association with unhealthy foods such as red meat, fast food, and high-fat diets (52) also underlines the validity and importance of this score.

## Conclusion

Consuming a diet rich in antioxidants determined by dTAC could decrease the risk of HNC cancer. Healthy dietary patterns which increase antioxidant intake should be encouraged, particularly in high-risk groups. These findings provided valuable insight for designing preventive policies such as promoting high antioxidant food intakes such as fruit and vegetable by subsidizing or providing free fruits in schools.

## Data availability statement

The original contributions presented in the study are included in the article/Supplementary material, further inquiries can be directed to the corresponding author.

## Ethics statement

The studies involving humans were approved by ethical committee of Tehran university of medical sciences. The studies were conducted in accordance with the local legislation and institutional requirements. Written informed consent for participation was not required from the participants or the participants’ legal guardians/next of kin in accordance with the national legislation and institutional requirements.

## Author contributions

FT and KZ designed the current study. KZ, EM, RS, FN, AN-T, MS, MH, PB, and HR designed and managed the data collection and cleaning of primary data (IROPICAN study). FT, BS, and HR performed the nutritional and statistical analysis. FT wrote the draft. All authors contributed to the article and approved the submitted version.

## References

[1] Ferlay J Colombet M Soerjomataram I Mathers C Parkin DM Piñeros M. Estimating the global cancer incidence and mortality in 2018: GLOBOCAN sources and methods. Int J Cancer. (2019) 144:1941–53. doi: 10.1002/ijc.31937, PMID: 30350310

[2] Ferlay J Colombet M Soerjomataram I Parkin DM Piñeros M Znaor A. Cancer statistics for the year 2020: an overview. Int J Cancer. (2021) 149:778–89. doi: 10.1002/ijc.33588 33818764

[3] Beeler WH Bellile EL Casper KA Jaworski E Burger NJ Malloy KM. Patient-reported financial toxicity and adverse medical consequences in head and neck cancer. Oral Oncol. (2020) 101:104521. doi: 10.1016/j.oraloncology.2019.104521, PMID: 31877502 PMC7008081

[4] Massa ST Osazuwa-Peters N Adjei Boakye E Walker RJ Ward GM. Comparison of the financial burden of survivors of head and neck cancer with other cancer survivors. JAMA Otolaryngol Head Neck Surg. (2019) 145:239–49. doi: 10.1001/jamaoto.2018.3982, PMID: 30789634 PMC6439752

[5] Patterson RH Fischman VG Wasserman I Siu J Shrime MG Fagan JJ. Global burden of head and neck cancer: economic consequences, health, and the role of surgery. Otolaryngol Head Neck Surg. (2020) 162:296–303. doi: 10.1177/0194599819897265, PMID: 31906785

[6] Fund WCR. Diet, nutrition, physical activity and cancers of the mount, pharynx and larynx. London: World Cancer Research Fund, American institute for Cancer Research. (2018).

[7] du E Mazul AL Farquhar D Brennan P Anantharaman D Abedi-Ardekani B. Long-term survival in head and neck cancer: impact of site, stage, smoking, and human papillomavirus status. Laryngoscope. (2019) 129:2506–13. doi: 10.1002/lary.27807, PMID: 30637762 PMC6907689

[8] Korn AR Reedy J Brockton NT Kahle LL Mitrou P Shams-White MM. The 2018 World Cancer Research Fund/American Institute for Cancer Research score and Cancer risk: a longitudinal analysis in the NIH-AARP diet and health study. Cancer Epidemiol Biomark Prev. (2022) 31:1983–92. doi: 10.1158/1055-9965.EPI-22-0044, PMID: 35877953 PMC9532348

[9] Aupérin A. Epidemiology of head and neck cancers: an update. Curr Opin Oncol. (2020) 32:178–86. doi: 10.1097/CCO.0000000000000629 32209823

[10] Piotrowski I Kulcenty K Suchorska W. Interplay between inflammation and cancer. Rep Pract Oncol Radiotherapy. (2020) 25:422–7. doi: 10.1016/j.rpor.2020.04.004, PMID: 32372882 PMC7191124

[11] Korniluk A Koper O Kemona H Dymicka-Piekarska V. From inflammation to cancer. Ir J Med Sci. (1971) 186:57–62. doi: 10.1007/s11845-016-1464-0, PMID: 27156054 PMC5323483

[12] Basu A Devaraj S Jialal I. Dietary factors that promote or retard inflammation. Arterioscler Thromb Vasc Biol. (2006) 26:995–1001. doi: 10.1161/01.ATV.0000214295.86079.d1 16484595

[13] Lee KW Lee HJ Lee CY. Vitamins, phytochemicals, diets, and their implementation in cancer chemoprevention. Crit Rev Food Sci Nutr. (2004) 44:437–52. doi: 10.1080/10408690490886674, PMID: 15615427

[14] Abbasalizad Farhangi M Vajdi M. Dietary total antioxidant capacity (TAC) significantly reduces the risk of site-specific cancers: an updated systematic review and meta-analysis. Nutr Cancer. (2021) 73:721–39. doi: 10.1080/01635581.2020.1771385, PMID: 32462920

[15] Griffiths K Aggarwal B Singh R Buttar H Wilson D de Meester F. Food antioxidants and their anti-inflammatory properties: a potential role in cardiovascular diseases and cancer prevention. Diseases. (2016) 4:28. doi: 10.3390/diseases4030028 28933408 PMC5456284

[16] Kim YS Young MR Bobe G Colburn NH Milner JA. Bioactive food components, inflammatory targets, and cancer prevention diet, inflammation, and cancer prevention. Cancer Prev Res. (2009) 2:200–8. doi: 10.1158/1940-6207.CAPR-08-0141 PMC3449301 19258539

[17] Khuda-Bukhsh AR Das S Saha SK. Molecular approaches toward targeted cancer prevention with some food plants and their products: inflammatory and other signal pathways. Nutr Cancer. (2014) 66:194–205. doi: 10.1080/01635581.2014.864420 24377653

[18] Freedman ND Park Y Subar AF Hollenbeck AR Leitzmann MF Schatzkin A. Fruit and vegetable intake and head and neck cancer risk in a large United States prospective cohort study. Int J Cancer. (2008) 122:2330–6. doi: 10.1002/ijc.23319, PMID: 18092323

[19] Suzuki T Wakai K Matsuo K Hirose K Ito H Kuriki K. Effect of dietary antioxidants and risk of oral, pharyngeal and laryngeal squamous cell carcinoma according to smoking and drinking habits. Cancer Sci. (2006) 97:760–7. doi: 10.1111/j.1349-7006.2006.00232.x, PMID: 16800818 PMC11159902

[20] Papadimitriou N Markozannes G Kanellopoulou A Critselis E Alhardan S Karafousia V. An umbrella review of the evidence associating diet and cancer risk at 11 anatomical sites. Nat Commun. (2021) 12:4579. doi: 10.1038/s41467-021-24861-8, PMID: 34321471 PMC8319326

[21] Hu FB. Dietary pattern analysis: a new direction in nutritional epidemiology. Curr Opin Lipidol. (2002) 13:3–9. doi: 10.1097/00041433-200202000-00002 11790957

[22] Nascimento-Souza MA Paiva PG Martino HSD Ribeiro AQ. Dietary total antioxidant capacity as a tool in health outcomes in middle-aged and older adults: a systematic review. Crit Rev Food Sci Nutr. (2018) 58:905–12. doi: 10.1080/10408398.2016.1230089, PMID: 27646047

[23] Pellegrini N Vitaglione P Granato D Fogliano V. Twenty-five years of total antioxidant capacity measurement of foods and biological fluids: merits and limitations. J Sci Food Agric. (2020) 100:5064–78. doi: 10.1002/jsfa.9550, PMID: 30578632

[24] Sheng L-T Jiang YW Pan A Koh WP. Dietary total antioxidant capacity and mortality outcomes: the Singapore Chinese health study. Eur J Nutr. (2022) 61:2375–82. doi: 10.1007/s00394-022-02812-3, PMID: 35122488

[25] Parohan M Sadeghi A Khatibi SR Nasiri M Milajerdi A Khodadost M. Dietary total antioxidant capacity and risk of cancer: a systematic review and meta-analysis on observational studies. Crit Rev Oncol Hematol. (2019) 138:70–86. doi: 10.1016/j.critrevonc.2019.04.003, PMID: 31092388

[26] Sasanfar B Toorang F Maleki F Esmaillzadeh A Zendehdel K. Association between dietary total antioxidant capacity and breast cancer: a case–control study in a middle eastern country. Public Health Nutr. (2021) 24:965–72. doi: 10.1017/S1368980019004397, PMID: 32234094 PMC10195604

[27] Hadji M Rashidian H Marzban M Gholipour M Naghibzadeh-Tahami A Mohebbi E. The Iranian study of opium and cancer (IROPICAN): Rationale, design, and initial findings. Arch Iran Med. (2021) 24:167–76. doi: 10.34172/aim.2021.27, PMID: 33878874

[28] Seyyedsalehi MS Collatuzzo G Huybrechts I Hadji M Rashidian H Safari-Faramani R. Association between dietary fat intake and colorectal cancer: a multicenter case-control study in Iran. Front Nutr. (2022) 9:1017720. doi: 10.3389/fnut.2022.1017720, PMID: 36466398 PMC9709886

[29] Mohebbi E Hadji M Rashidian H Rezaianzadeh A Marzban M Haghdoost AA. Opium use and the risk of head and neck squamous cell carcinoma. Int J Cancer. (2021) 148:1066–76. doi: 10.1002/ijc.33289, PMID: 32895947

[30] Kauppinen T Heikkilä P Plato N Woldbæk T lenvik Hansen J. Construction of job-exposure matrices for the Nordic occupational cancer study (NOCCA). Acta Oncol. (2009) 48:791–800. doi: 10.1080/02841860902718747, PMID: 19225948

[31] Poustchi H Eghtesad S Kamangar F Etemadi A Keshtkar AA Hekmatdoost A. Prospective epidemiological research studies in Iran (the PERSIAN cohort study): rationale, objectives, and design. Am J Epidemiol. (2018) 187:647–55. doi: 10.1093/aje/kwx314, PMID: 29145581 PMC6279089

[32] USDA National Nutrient Database for Standard Reference (2021). Agriculture UDo USDA food composition 2018. Available at: https://fdc.nal.usda.gov/ (Accesed September 11, 2022).

[33] Pellegrini N Colombi B del Rio D Salvatore S Bianchi M Brighenti F. Total antioxidant capacity of plant foods, beverages and oils consumed in Italy assessed by three different in vitro assays. J Nutr. (2003) 133:2812–9. doi: 10.1093/jn/133.9.2812, PMID: 12949370

[34] Pellegrini N Serafini M Salvatore S del Rio D Bianchi M Brighenti F. Total antioxidant capacity of spices, dried fruits, nuts, pulses, cereals and sweets consumed in Italy assessed by three different in vitro assays. Mol Nutr Food Res. (2006) 50:1030–8. doi: 10.1002/mnfr.200600067, PMID: 17039458

[35] Hu FB Stampfer MJ Rimm E Ascherio A Rosner BA Spiegelman D. Dietary fat and coronary heart disease: a comparison of approaches for adjusting for total energy intake and modeling repeated dietary measurements. Am J Epidemiol. (1999) 149:531–40. doi: 10.1093/oxfordjournals.aje.a009849, PMID: 10084242

[36] Chuang S-C Jenab M Heck JE Bosetti C Talamini R Matsuo K. Diet and the risk of head and neck cancer: a pooled analysis in the INHANCE consortium. Cancer Causes Control. (2012) 23:69–88. doi: 10.1007/s10552-011-9857-x, PMID: 22037906 PMC3654401

[37] Abdi F. Atarodi Zahra Mirmiran P. Esteki T. (2015). Surveying global and Iranian food consumption patterns: a review of the literature.

[38] Lam TK Cross AJ Freedman N Park Y Hollenbeck AR Schatzkin A. Dietary fiber and grain consumption in relation to head and neck cancer in the NIH-AARP diet and health study. Cancer Causes Control. (2011) 22:1405–14. doi: 10.1007/s10552-011-9813-9, PMID: 21785948 PMC3215506

[39] Li W-Q Park Y Wu JW Goldstein AM Taylor PR Hollenbeck AR. Index-based dietary patterns and risk of head and neck cancer in a large prospective study. Am J Clin Nutr. (2014) 99:559–66. doi: 10.3945/ajcn.113.073163, PMID: 24401718 PMC3927689

[40] Bravi F Edefonti V Randi G Ferraroni M la Vecchia C Decarli A. Dietary patterns and upper aerodigestive tract cancers: an overview and review. Ann Oncol. (2012) 23:3024–39. doi: 10.1093/annonc/mds197, PMID: 22967993

[41] Mozaffari H Daneshzad E Surkan PJ Azadbakht L. Dietary total antioxidant capacity and cardiovascular disease risk factors: a systematic review of observational studies. J Am Coll Nutr. (2018) 37:533–45. doi: 10.1080/07315724.2018.1441079, PMID: 29714643

[42] Fearon IM Phillips G Carr T Taylor M Breheny D Faux SP. The role of oxidative stress in smoking-related diseases. Mini-Rev Org Chem. (2011) 8:360–71. doi: 10.2174/157019311797440317

[43] Das SK Vasudevan D. Alcohol-induced oxidative stress. Life Sci. (2007) 81:177–87. doi: 10.1016/j.lfs.2007.05.005 17570440

[44] Leoncini E Edefonti V Hashibe M Parpinel M Cadoni G Ferraroni M. Carotenoid intake and head and neck cancer: a pooled analysis in the international head and neck cancer epidemiology consortium. Eur J Epidemiol. (2016) 31:369–83. doi: 10.1007/s10654-015-0036-3, PMID: 25930054 PMC5552068

[45] Bondonno NP Dalgaard F Kyrø C Murray K Bondonno CP Lewis JR. Flavonoid intake is associated with lower mortality in the Danish diet cancer and health cohort. Nat Commun. (2019) 10:3651. doi: 10.1038/s41467-019-11622-x, PMID: 31409784 PMC6692395

[46] Malathi M Vijay M Shivashankara A. The role of oxidative stress and the effect of radiotherapy on the plasma oxidant-antioxidant status in head and neck cancer. J Clin Diagn Res. (2011) 5:249–51.

[47] Ferraguti G Terracina S Petrella C Greco A Minni A Lucarelli M. Alcohol and head and neck cancer: updates on the role of oxidative stress, genetic, epigenetics, oral microbiota, antioxidants, and alkylating agents. Antioxidants. (2022) 11:145. doi: 10.3390/antiox11010145, PMID: 35052649 PMC8773066

[48] Singh A Pandey P Tewari M Pandey HP Gambhir IS Shukla HS. Free radicals hasten head and neck cancer risk: a study of total oxidant, total antioxidant, DNA damage, and histological grade. J Postgrad Med. (2016) 62:96–101. doi: 10.4103/0022-3859.180555, PMID: 27089108 PMC4944358

[49] Bairati I Meyer F Gelinas M Fortin A Nabid A Brochet F. A randomized trial of antioxidant vitamins to prevent second primary cancers in head and neck cancer patients. J Natl Cancer Inst. (2005) 97:481–8. doi: 10.1093/jnci/dji095, PMID: 15812073

[50] Bairati I Meyer F Gélinas M Fortin A Nabid A Brochet F. Randomized trial of antioxidant vitamins to prevent acute adverse effects of radiation therapy in head and neck cancer patients. J Clin Oncol. (2005) 23:5805–13. doi: 10.1200/JCO.2005.05.514 16027437

[51] Guerra J Devesa J. Usefulness of melatonin and other compounds as antioxidants and epidrugs in the treatment of head and neck cancer. Antioxidants. (2021) 11:35. doi: 10.3390/antiox11010035, PMID: 35052539 PMC8773331

[52] Puchau B Zulet MÁ de Echávarri AG Hermsdorff HHM Martínez JA. Dietary total antioxidant capacity: a novel indicator of diet quality in healthy young adults. J Am Coll Nutr. (2009) 28:648–56. doi: 10.1080/07315724.2009.10719797 20516264

[53] Pitsavos C Panagiotakos DB Tzima N Chrysohoou C Economou M Zampelas A. Adherence to the Mediterranean diet is associated with total antioxidant capacity in healthy adults: the ATTICA study. Am J Clin Nutr. (2005) 82:694–9. doi: 10.1093/ajcn/82.3.694 16155285

