# Supplementary material for: Dietary total antioxidant capacity and head and neck cancer: a large case-control study in Iran
Source: Front Nutr. 2023 Sep 27;10:1226446. doi: 10.3389/fnut.2023.1226446 (PMC10569465; doi:10.3389/fnut.2023.1226446)
Supplement: Supplementary file 1 [file Table_1.pdf]

Supplementary Table 1.The association of dietary Total Antioxidant Capacity (dTAC) scores with head and neck squamous cell carcinoma in different groups

	FRAP[2]					TRAP[3]				
	Cases	OR (95%CI)	Cases	OR (95%CI)		Cases	OR (95%CI)	Cases	OR (95%CI)	
Smoking		No		Yes	P		No		Yes	P
First tertile	167	Reference	194	1.5(1.1-2.0)	0.06	151	Reference	239	2.1(1.6-2.9)	0.41
Second tertile	118	0.54(0.41-0.71)	143	1.1(0.83-1.6)		112	0.55(0.41-0.74)	122	0.9(0.65-1.3)	
Third tertile	97	0.39(0.29-0.53)	157	0.97(0.7-1.3)		119	0.53(0.39-0.71)	133	0.98(0.69-1.4)	
Opium use		No		Yes	P		No		Yes	P
First tertile	195	Reference	166	3.9(2.9-5.4)	0.67	179	Reference	211	4.77(3.5-6.5)	0.27
Second tertile	141	0.63(0.49-0.51)	120	2.4(1.7-3.4)		141	0.54(0.42-0.71)	93	1.8(1.3-2.6)	
Third tertile	138	0.46(0.36-0.61)	116	2.2(1.5-3.1)		154	0.52(0.39-0.68)	98	2.2(1.5-3.1)	
Water pipe use		No		Yes	P		No		Yes	P
First tertile	341	Reference	20	1.2(0.63-2.1)	0.38	368	Reference	22	0.89(0.51-1.6)	0.42
Second tertile	234	0.62(0.49-0.77)	27	0.86(0.5-1.5)		205	0.47(0.37-0.59)	29	0.66(0.39-1.1)	
Third tertile	221	0.51(0.4-0.65)	33	0.44(0.27-0.69)		223	0.49(0.39-0.63)	29	0.47(0.29-0.78)	
Alcohol use		No		Yes	P		No		Yes	P
First tertile	336	Reference	25	0.71(0.41-1.2)	0.51	362	Reference	28	0.72(0.41-1.2)	0.45
Second tertile	239	0.61(0.49-0.76)	22	0.64(0.35-1.2)		213	0.48(0.38-0.60)	21	0.43(0.23-0.79)	
Third tertile	230	0.48(0.38-0.60)	24	0.52(0.29-0.95)		230	0.48(0.38-0.60)	22	0.58(0.31-1.1)	

Sex		Women		Men	p		Women		Men	p
First tertile	88	Reference	273	0.81(0.55-1.2)	0.96	91	Reference	299	0.87(0.59-1.3)	0.89
Second tertile	71	0.60(0.42-0.87)	190	0.51(0.34-0.77)		59	0.48(0.32-0.71)	175	0.42(0.28-0.63)	
Third tertile	55	0.47(0.32-0.71)	199	0.40(0.27-0.60)		64	0.52(0.35-0.77)	188	0.42(0.28-0.63)	
Age		Younger than 50		Older than 50	p		Younger than 50		Older than 50	p
First tertile	85	Reference	276	0.79(0.57-1.1)	0.26	83	Reference	307	0.73(0.53-1.0)	0.59
Second tertile	61	0.85(0.56-1.3)	200	0.45(0.32-0.64)		53	0.59(0.38-0.91)	181	0.34(0.38-0.91)	
Third tertile	48	0.57(0.36-0.89)	206	0.37(0.26-0.53)		58	0.55(0.36-0.84)	194	0.34(0.24-0.49)	

[1]Adjusted for energy (continues, Kcal/d), age(five categories) and sex (male/female), socioeconomic status (low, medium, high), smoking (yes, no), water pipe use (yes, no), regular alcohol use (yes, no), physical activity (sedentary, moderate, heavy, unknown) and dental health(poor, moderate, good)
[2]FRAP: Ferric Reducing Antioxidant Power of the diet
[3]TRAP: Total Radical-trapping Antioxidan**t** Parameters of the diet
